# Modeling Peptide–Protein Interactions by a Logo-Based Method: Application in Peptide–HLA Binding Predictions

**DOI:** 10.3390/molecules29020284

**Published:** 2024-01-05

**Authors:** Irini Doytchinova, Mariyana Atanasova, Antonio Fernandez, F. Javier Moreno, Frits Koning, Ivan Dimitrov

**Affiliations:** 1 Faculty of Pharmacy, Medical University of Sofia, 1000 Sofia, Bulgaria; matanasova@pharmfac.mu-sofia.bg (M.A.); idimitrov@pharmfac.mu-sofia.bg (I.D.); 2 European Food Safety Authority, 43126 Parma, Italy; antonio.fernandezdumont@efsa.europa.eu; 3 Instituto de Investigación en Ciencias de la Alimentación (CIAL), CSIC-UAM, CEI (UAM+CSIC), Nicolás Cabrera, 9, 28049 Madrid, Spain; javier.moreno@csic.es; 4 Department of Immunohematology and Blood Transfusion, Leiden University Medical Centre, 2333 ZA Leiden, The Netherlands; f.koning@lumc.nl

**Keywords:** peptide–protein interactions, logo method, HLA-DQ2.5, HLA-DQ8.1

## Abstract

Peptide–protein interactions form a cornerstone in molecular biology, governing cellular signaling, structure, and enzymatic activities in living organisms. Improving computational models and experimental techniques to describe and predict these interactions remains an ongoing area of research. Here, we present a computational method for peptide–protein interactions’ description and prediction based on leveraged amino acid frequencies within specific binding cores. Utilizing normalized frequencies, we construct quantitative matrices (QMs), termed ‘logo models’ derived from sequence logos. The method was developed to predict peptide binding to HLA-DQ2.5 and HLA-DQ8.1 proteins associated with susceptibility to celiac disease. The models were validated by more than 17,000 peptides demonstrating their efficacy in discriminating between binding and non-binding peptides. The logo method could be applied to diverse peptide–protein interactions, offering a versatile tool for predictive analysis in molecular binding studies.

## 1. Introduction

Peptide–protein interactions are among the main fundaments in molecular biology, underlying a diverse array of cellular functions like cellular signaling, structural organization, and enzymatic activities within living organisms. These interactions are based on electrostatic attractions and repulsions, hydrogen bonding, van der Waals forces, and hydrophobic effects and they determine the affinity between the molecules and the stability of peptide–protein complexes [1]. These interactions not only dictate the folding and assembly of proteins but also play a crucial role in modulating their function. For example, the binding of the peptide insulin to its receptor regulates glucose metabolism [2]. Chaperone proteins, assisted by peptide interactions, facilitate the correct folding of newly synthesized proteins and prevent misfolding, contributing to cellular homeostasis [3]. Peptide-based therapeutics, such as peptide inhibitors or mimetics, target specific protein–protein interactions associated with diseases, offering promising opportunities for novel drug design and development [4].

Advancements in experimental techniques have significantly contributed to unraveling the complexities of peptide–protein interactions. Nuclear Magnetic Resonance (NMR) spectroscopy, X-ray crystallography, and cryo-electron microscopy provide detailed structural insights into these interactions at the atomic level, elucidating binding interfaces and conformational changes [5]. Additionally, computational approaches, including molecular dynamics simulations and docking studies, complement experimental data, facilitating the prediction and understanding of peptide–protein interactions in silico [6].

Here, we present a computational method for quantitative description of peptide–protein interactions based on amino acid frequencies at each of the nine positions from the peptide binding core. The normalized frequencies enter a quantitative matrix (QM). Because the amino acid frequencies for a given binding motif are presented by a sequence logo [7], we named the QMs derived by this method ‘logo models’. We describe the development of logo models for the peptide binding prediction of the proteins HLA-DQ2.5 and HLA-DQ8.1. HLA-DQ2 and HLA-DQ8 proteins play a pivotal role in presenting gluten fragments to immune cells, setting off an immune response in individuals afflicted with celiac disease. These proteins engage with gluten peptides, facilitating their presentation to T cells, which in turn triggers the activation of the immune system and ultimately leads to damage of the small intestine [8,9]. The peptide binding sites within HLA-DQ2 and HLA-DQ8 are remarkably well-preserved binding grooves created to accommodate peptide binding cores comprised of nine amino acids with specific compositions. The assessment of novel proteins for their potential as HLA-DQ2 and/or HLA-DQ8 binders carries profound significance in the development of secure and nourishing products accessible to all consumers, including those managing celiac disease. The ability of the logo models to recognize peptides binding to these two alleles was evaluated by external test sets. Although the logo method was developed to predict peptide binding to DQ proteins, it is universal and can be applied to any peptide–protein interaction.

## 2. Results

### 2.1. Sequence Logo-Based Model for Peptide Binding Prediction for HLA-DQ2.5

The training and test datasets contain known binders to HLA-DQ proteins (positive sets) and non-binders (negative sets). The training sets were used for the development of logo models. The test sets were used for the validation of the derived models. 

The training set of binders to HLA-DQ2.5 was derived from Stepniak et al. [10]. It consisted of 125 nonamer binding cores. Despite our diligent efforts to curate a reliable negative set comprising experimentally validated non-binders sourced from various databases and references, we encountered challenges and were unable to derive such a set. To tackle this challenge, we devised an alternative approach by creating a set of non-binders. This involved considering the total combination of non-preferred amino acids across all nine positions within the binding core, according to the known binding motifs for DQ2.5 [11]. Given that the peptides encompass experimentally confirmed non-preferred amino acids at each position, the combination of these specific amino acids inevitably yields sequences classified as non-binders. The non-preferred amino acids for DQ2.5 are as follows: for position 1 (p1)—Ala, Arg, Asn, Asp, Glu, Gly, Lys, Ser, and Thr; for position 2 (p2)—Ala and Leu; for position 3 (p3)—Arg, Asp, Glu, Ile, Leu, and Lys; for position 4 (p4)—Ala, Arg, Gln, Gly, Ile, Leu, Lys, Met, Phe, Ser, Thr, Trp, and Val; for position 5 (p5)—Arg, Leu, Lys, and Thr; for position 6 (p6)—Arg, Asn, Gln, Gly, Leu, Lys, Pro, Ser, Thr, and Val; for position 7 (p7)—Ala, Asn, Gln, Gly, Leu, Lys, Pro, Ser, Thr, and Val; for position 8 (p8)—Ala, Gln, Ile, Leu, and Thr; for position 9 (p9)—Ala, Arg, Gln, Lys, Met, Pro, Ser, and Thr [11]. The total combination of them generated a pool of 24,710,400 non-binding nonamers. Among them, a set of 125 nonamers was randomly selected and used as a negative training set for the development of the logo model for DQ2.5. 

The test set of binders to DQ2.5 was obtained from LC-MS data, comprising 4249 binders of varying lengths [12]. The same number of non-binding nonamers was randomly chosen from the non-binders pool, including nonamers distinct from those in the negative training set used for model development. Table 1 presents a summary of the peptides utilized in this study.

The amino acid frequencies at each position within a nonamer are normalized by their mean using the following formula:

$$X_{i}\left(norm\right)=\frac{X_{i}-X_{mean}}{X_{max}-X_{min}}$$

where *X_i_* is the frequency of amino acid *i* at a given position, *X_mean_* is the mean frequency at the same position, and *X_max_* and *X_min_* are the maximum and the minimum frequencies, respectively, at the same position. The normalized values fall in the range [−1, 1].

**Figure 1:**
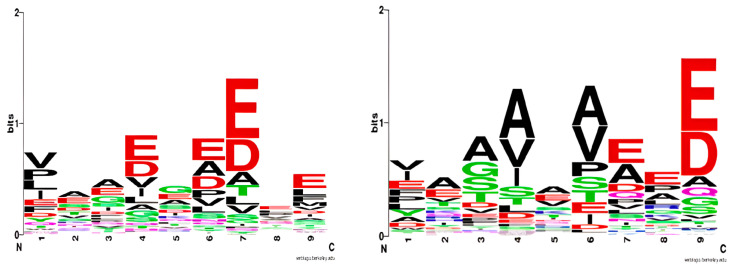
Sequence logo for HLA-DQ2.5 based on a training set of 125 binding nonamers (**left**); sequence logo for HLA-DQ8.1 based on a training set of 463 binding nonamers (**right**). The sequence logos were generated by WebLogo (https://weblogo.berkeley.edu, accessed on 11 April 2022).

The normalized values are organized into a quantitative matrix (QM) measuring 9 positions by 20 amino acids (Table 2). This QM is termed a ‘logo model,’ inspired by the graphical representation of amino acid sequence conservation in proteins [7]. In the sequence logo, each position is represented by a stack of letters, where the size of the letters reflects their frequency within the sequences. Figure 1 (left) presents the sequence logo for HLA-DQ2.5, derived from the training set comprising 125 binding nonamers.

We further develop this method by quantifying the amino acid frequencies at each position of the binding peptide. In the logo model, the size of the letters is substituted by positive and negative quantitative values (Table 2). The positive values correspond to preferred amino acids at a given peptide position, and the negative values correspond to non-preferred ones.

Similarly, a logo model was constructed for the set of non-binders (Table 3). The two logo models were used to calculate the binding (BSs) and non-binding scores (NBSs) of a tested peptide by summarizing the values of the corresponding amino acids at each position. If BS is higher than NBS, the nonamer is classified as a binder. Otherwise, it is a non-binder.

The predictive ability of the logo models was tested on an external test set consisting of 4249 binders and 4249 non-binders to HLA-DQ2.5. The performance of the logo models is given in Table 4. They recognize 90% of the binders and 100% of the non-binders. 

**Figure 2:**
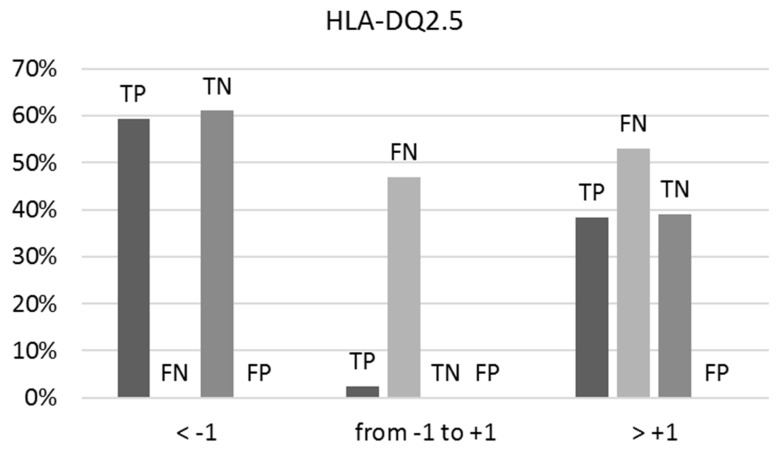
The percentages of true positives (TPs), false negatives (FNs), true negatives (TNs), and false positives (FPs) vary concerning the ranges of the BS/NBS ratio for binders and NBS/BS ratio for non-binders to HLA-DQ2.5, as indicated by predictions on the external test set. Optimal predictions occur at ratios below −1 and above +1.

When the BS/NBS ratio for the binders and the NBS/BS ratio for the non-binders approach unity (between −1 and +1), the prediction uncertainty escalates, leading to a 47% occurrence of false negatives (FNs) as depicted in Figure 2. Within the true positive (TP) category, this uncertainty remains low at only 2%. Among true negatives (TNs) and false positives (FPs), it diminishes further to 0%. Optimal predictions occur at ratios below −1 and above +1.

### 2.2. Sequence Logo-Based Model for Peptide Binding Prediction to HLA-DQ8.1

A set of 463 strong binding nonamers was selected from Tran et al. [13] and used to derive the sequence logo for DQ8.1 (Figure 1 right). The logo derived in the present study is in good agreement with Nielsen’s logo [14]. The only difference is the preference for Val and Ile at anchor p1, followed by Glu, according to our model. The p1 pocket is deep and polar, lined by two positively charged residues His24 and Arg52 and two negatively charged ones Glu31 and Glu86 [15]. Henderson et al. [15] has shown that Glu at p1 forms two salt bridges with Arg52 and a water-mediated network with His24, Glu31, and Arg53. The preference for the hydrophobic Val and Ile at p1 is a novel observation for HLA-DQ8.1. 

Similarly to the non-binder set for HLA-DQ2.5, the non-binder set for HLA-DQ8.1 was assembled by combining non-preferred amino acids across all nine positions within the binding core, aligning with the established binding motif for DQ8.1 [13]. The non-preferred amino acids are the following: for position 1 (p1)—Arg, Asn, Cys, Gln, Gly, His, Leu, Lys, Ser, and Thr; for position 2 (p2)—Cys, Leu, Phe, and Pro; for position 3 (p3)—Asn, Arg, Cys, Gln, Glu, His, Ile, Leu, and Lys; for position 4 (p4)—Arg, Asn, Glu, Gln, Gly, His, Leu, Lys, Phe, Pro, and Tyr; for position 5 (p5)—Asn, Cys, and Leu; for position 6 (p6)—Asn, Arg, Cys, Gln, Gly, His, Leu, Lys, Phe, and Tyr; for position 7 (p7)—Arg, Cys, Gly, Leu, Lys, Met, and Thr; for position 8 (p8)—Asn, Cys, and Leu; and for position 9 (p9)—Arg, Asn, Cys, Ile, Leu, Lys, Phe, Pro, Ser, Thr, and Val [13]. The pool of non-binders contained 27,442,800 nonamers. From them, a set of 463 nonamers was randomly selected and used for the development of the logo model for non-binders to DQ8.1.

The normalized amino acid frequencies for binders and non-binders enter the corresponding logo models (Table 5 and Table 6). Their predictive ability was tested on an external test set. The test set of binders to HLA-DQ8.1 were collected from LC-MS data and contained 4339 known strong binders [13]. An equivalent quantity of non-binding nonamers was randomly chosen from the non-binder pool, comprising nonamers distinct from those present in the negative training set. The performance is given in Table 4. They recognize 98% of the binders and 100% of the non-binders. 

**Figure 3:**
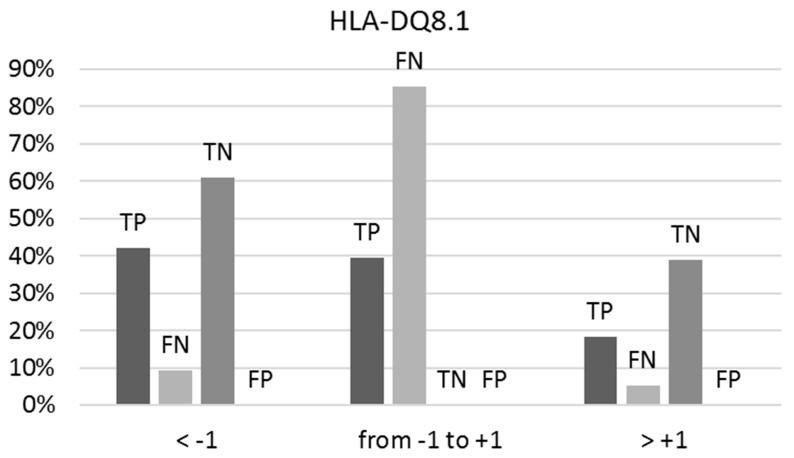
The percentages of true positives (TPs), false negatives (FNs), true negatives (TNs), and false positives (FPs) vary concerning the ranges of the BS/NBS ratio for binders and NBS/BS ratio for non-binders to HLA-DQ8.1, as indicated by predictions on the external test set. Optimal predictions occur at ratios below −1 and above +1.

Here again, like the predictions for HLA-DQ2.5, uncertainty in prediction intensifies when both the BS/NBS and NBS/BS ratios approximate unity (between −1 and +1). Among the true positives, 40% show a BS/NBS ratio within this range, while 85% of the false negatives are within this range (Figure 3). All true negatives consistently present an NBS/BS ratio below −1, and no instances of false positives have been observed.

## 3. Discussion

In the present study, we introduce a new method for evaluating and predicting interactions between peptides and proteins that can be applied universally. The method employs a scoring system based on amino acid frequencies at specific positions in binding and non-binding peptides. We trained the method using datasets of known binders and non-binders to HLA-DQ2.5 and HLA-DQ8.1 and evaluated its performance on external test sets of more than 17,000 peptides. The results demonstrated that our approach achieved a high level of accuracy in predicting peptide binding to these two proteins. The derived models could be applied for the in silico search of peptides binding to HLA-DQ2.5 or/and HLA-DQ8.1, according to the European Food Safety Authority’s (EFSA) guidance on the risk assessment of novel proteins and their capacity to cause celiac disease [16]. The guidance outlines a framework for assessing the potential risk posed by new proteins introduced into the food supply, including the use of in silico, in vitro, and in vivo methods to evaluate their potential for triggering an immune response. According to this guidance, initially, the tested protein is compared to known proteins associated with celiac disease and if any identity or similarity is observed, then the protein is searched for binders to HLA-DQ2.5 or/and HLA-DQ8.1. If strong peptide binders to HLA-DQ2.5 and DQ8.1 are predicted, then HLA-DQ binding assays are performed to confirm or reject these predictions together with in vitro digestibility tests and/or tests for identification of T-cell epitopes. The guidance ensures the safety of novel proteins in the food supply and provides a useful tool for food manufacturers and regulatory agencies to evaluate the risk of celiac disease associated with new proteins.

The main binding motif for HLA-DQ2.5 includes bulky hydrophobic residues at positions p1 and p9 and negatively charged residues at positions p4, p6, and/or p7 [17,18]. Stepniak et al. have defined another binding motif consisting of proline or polar residues at p1; acidic or polar residues at p4, p6, and p7; and hydrophobic or polar residues at p9 [10]. Koşaloğlu-Yalçın et al. analyzed peptides eluted by high-throughput mass spectroscopy and found that 75% of the HLA-DQ2.5 binders did not conform to any known binding motif [11]. They conclude that HLA-DQ2.5 can bind peptides promiscuously using alternate modes.

The binding motif for HLA-DQ2.5 obtained in the present study is compatible with Stepniak’s definition because the positive training set used to derive the logo model for DQ2.5 binders was compiled from their paper [10]. Nevertheless, the model recognized 90% of 4249 binders from the external test set. We attribute this high predictive ability to the unique selection of the negative set, which has been defined in a novel way.

The binding motif for HLA-DQ8.1 is more consistent. Specifically, HLA-DQ8.1 prefers peptides that contain negatively charged or polar residues at positions p1, p7, p8, and p9 and small aliphatic residues at positions p4 and p6 [13]. The predictive ability of the logo models here is even higher—99% accuracy for a test set of 8678 peptides. Again, this is due to the powerful combinatorial library of non-binders generated from the non-preferred amino acids at all nine positions of the binding core.

## 4. Datasets and Methods

### 4.1. Training and Test Datasets

The HLA-DQ2.5 positive training set, sourced from Stepniak et al. [10], comprised 125 nonamer binding cores. From Tran et al. [13], a positive training set of 463 binding nonamers to HLA-DQ8.1 was selected. To generate sets of non-binders, we combined non-preferred amino acids across all nine positions based on known binding motifs for DQ2.5 [11] and DQ8.1 [13].

From the pool of DQ2.5 non-binders, a subset of 125 nonamers was randomly chosen as a negative training set for the development of the DQ2.5 logo model. Similarly, for DQ8.1, a subset of 463 nonamers was randomly selected for the development of the DQ8.1 logo model from the non-binder pool.

The test sets comprising binders were compiled from LC-MS data. For HLA-DQ2.5, it encompassed 4249 binders of varying lengths [12], while for HLA-DQ8.1, it comprised 4339 known strong binders [13]. To ensure unbiased testing, an equivalent number of non-binding nonamers were randomly drawn from the non-binder pool, excluding those from the negative training sets used in model development.

### 4.2. Measures of Validation Accuracy

The assessment of the predictive capability of the logo models developed in this study involved evaluating *sensitivity*, *specificity,* and *accuracy*. *Sensitivity* gauges the accurate prediction of binding peptides and is computed using the formula:

$$Sensitivity=\frac{true\;positives}{true\;positives+false\;negatives}$$

*Specificity* measures the accurate prediction of non-binding peptides and is calculated using the formula:

$$Specificity=\frac{true\;negatives}{true\;negatives+false\;positives}$$

*Accuracy* reflects the overall performance of the models and is calculated using the formula:

$$Accuracy=\frac{true\;positives+true\;negatives}{true\;positives+true\;negatives+false\;positives+false\;negatives}$$

True positives represent peptides correctly predicted as binders, while true negatives are peptides correctly identified as non-binders. False positives refer to non-binders incorrectly predicted as binders, and false negatives are non-binders inaccurately predicted as binders. 

## 5. Conclusions

The method proposed in this study was developed especially for two HLA-DQ proteins associated with celiac disease; however, it has broad applicability. It is appropriate for antigen-antibody recognition, enzyme–substrate binding, peptide hormone–receptor interaction, protein–protein interaction domains, ion channel modulation by peptides, etc. The method takes a set of experimental data for equal-length peptides that either bind or do not bind to a given protein as input. The frequency of amino acids at each position in the binders is normalized and used to create a QM. A QM for non-binders is compiled in the same way. These QMs are then used to calculate BS and NBS for each peptide. Peptides are classified as binders if their BS is higher than their NBS and as non-binders otherwise. A significant advantage of the method is the absence of score ranking and a predefined threshold. It is intuitive, easy to develop without requiring specific software, and provides highly accurate predictions with unambiguous interpretations. The approach developed for assessing the potential of a novel protein to cause celiac disease can inspire the use of in silico predictive models targeting other properties relevant in the safety assessment of proteins.

## Figures and Tables

**Table 1:** Number of peptides in the training and test sets used in the study.

Datasets	Training Set	Test Set
**HLA-DQ2.5**		
Binders (positive set)	125 ^1^	4249 ^3^
Non-binders (negative set)	125 ^2^	4249 ^2^
**HLA-DQ8.1**		
Binders (positive set)	463 ^4^	4339 ^4^
Non-binders (negative set)	463 ^4^	4339 ^4^

^1^ [10]; ^2^ [11]; ^3^ [12]; ^4^ [13].

**Table 2:** Quantitative matrix (QM) for binding nonamers to HLA-DQ2.5.

AA	p1	p2	p3	p4	p5	p6	p7	p8	p9
Ala	−0.109	0.245	0.286	0.036	0.161	0.286	0.099	0.057	−0.005
Arg	−0.130	−0.047	−0.109	−0.130	−0.047	−0.130	−0.130	−0.068	−0.109
Asn	−0.089	−0.005	−0.068	−0.068	−0.047	−0.047	−0.130	−0.109	−0.089
Asp	0.016	0.099	0.016	0.286	0.057	0.224	0.411	0.036	0.016
Cys	−0.130	−0.130	−0.109	−0.130	−0.130	−0.130	−0.130	−0.130	−0.130
Gln	−0.005	−0.026	−0.005	−0.047	0.016	−0.047	−0.089	0.036	−0.068
Glu	0.078	0.224	0.224	0.536	0.182	0.474	0.870	0.224	0.495
Gly	−0.089	0.036	0.203	0.036	0.286	−0.068	−0.068	−0.068	−0.047
His	−0.130	−0.109	−0.130	−0.109	−0.130	−0.130	−0.130	−0.047	−0.109
Ile	0.161	−0.026	0.057	0.099	0.036	−0.089	−0.089	−0.005	0.016
Leu	0.182	−0.089	−0.005	0.057	−0.005	0.057	0.016	0.141	0.182
Lys	−0.130	−0.005	−0.130	−0.130	0.016	−0.130	−0.130	−0.047	−0.130
Met	−0.089	−0.089	−0.109	−0.130	−0.130	−0.109	−0.130	−0.089	0.057
Phe	0.078	−0.109	−0.005	−0.089	−0.089	−0.109	−0.130	−0.068	0.141
Pro	0.224	−0.089	−0.005	−0.109	−0.109	0.099	−0.089	0.141	−0.068
Ser	−0.130	0.120	0.078	0.016	0.099	0.016	−0.026	−0.005	−0.026
Thr	−0.047	0.099	−0.047	−0.047	−0.005	−0.068	0.057	0.016	−0.047
Trp	−0.068	−0.109	−0.130	−0.130	−0.089	−0.130	−0.109	−0.089	−0.068
Tyr	0.016	−0.068	−0.005	−0.109	−0.068	−0.068	−0.089	−0.026	−0.047
Val	0.391	0.078	−0.005	0.161	−0.005	0.099	0.016	0.099	0.036

**Table 3:** Quantitative matrix (QM) for non-binding nonamers to HLA-DQ2.5.

AA	p1	p2	p3	p4	p5	p6	p7	p8	p9
Ala	−0.019	0.799	−0.095	0.042	−0.095	−0.095	0.057	0.239	0.133
Arg	0.057	−0.095	0.148	0.148	0.572	0.133	0.178	−0.095	0.133
Asn	0.133	−0.095	−0.095	−0.095	−0.095	0.208	−0.034	−0.095	−0.095
Asp	0.178	−0.095	0.208	−0.095	−0.095	−0.095	−0.095	−0.095	−0.095
Cys	−0.095	−0.095	−0.095	−0.095	−0.095	−0.095	−0.095	−0.095	−0.095
Gln	−0.095	−0.095	−0.095	0.027	−0.095	0.117	0.027	0.193	0.148
Glu	0.133	−0.095	0.208	−0.095	−0.095	−0.095	−0.095	−0.095	−0.095
Gly	0.284	−0.095	−0.095	0.087	−0.095	0.208	0.102	−0.095	−0.095
His	−0.095	−0.095	−0.095	−0.095	−0.095	−0.095	−0.095	−0.095	−0.095
Ile	−0.095	−0.095	0.269	0.042	−0.095	−0.095	−0.095	0.375	−0.095
Leu	−0.095	0.905	0.193	0.057	0.345	0.027	0.057	0.254	−0.095
Lys	0.011	−0.095	0.299	−0.019	0.375	0.042	0.087	−0.095	0.027
Met	−0.095	−0.095	−0.095	0.042	−0.095	−0.095	−0.095	−0.095	0.223
Phe	−0.095	−0.095	−0.095	−0.004	−0.095	−0.095	−0.095	−0.095	−0.095
Pro	−0.095	−0.095	−0.095	−0.095	−0.095	0.042	0.027	−0.095	0.057
Ser	0.087	−0.095	−0.095	0.011	−0.095	0.072	0.102	−0.095	0.299
Thr	0.178	−0.095	−0.095	0.148	0.223	0.042	0.117	0.360	0.117
Trp	−0.095	−0.095	−0.095	0.133	−0.095	−0.095	−0.095	−0.095	−0.095
Tyr	−0.095	−0.095	−0.095	−0.095	−0.095	−0.095	−0.095	−0.095	−0.095
Val	−0.095	−0.095	−0.095	−0.049	−0.095	0.057	0.133	−0.095	−0.095

**Table 4:** Predictive ability of the logo models for HLA-DQ2.5 and HLA-DQ8.1 tested on external test sets. If the binding score (BS) is higher than the non-binding score (NBS), the nonamer is classified as a binder. Otherwise, it is a non-binder.

Parameter	HLA-DQ2.5	HLA-DQ8.1
Sensitivity %	90	98
Specificity %	100	100
Accuracy %	95	99

**Table 5:** Quantitative matrix (QM) for binding nonamers to HLA-DQ8.1.

AA	p1	p2	p3	p4	p5	p6	p7	p8	p9
Ala	0.068	0.373	0.494	0.699	0.262	0.531	0.378	0.152	0.031
Arg	−0.111	0.004	−0.085	−0.122	−0.027	−0.122	−0.111	0.047	−0.096
Asn	−0.122	−0.053	−0.106	−0.096	−0.074	−0.117	−0.038	−0.080	−0.106
Asp	0.036	−0.038	0.020	−0.011	−0.022	−0.038	0.083	−0.038	0.489
Cys	−0.122	−0.122	−0.122	−0.090	−0.122	−0.122	−0.122	−0.122	−0.122
Gln	−0.106	0.004	−0.090	−0.106	−0.022	−0.111	0.052	−0.074	0.010
Glu	0.157	0.215	−0.022	−0.053	0.194	0.062	0.494	0.383	0.878
Gly	−0.106	−0.053	0.257	−0.074	−0.032	−0.111	−0.090	0.041	0.004
His	−0.101	−0.006	−0.096	−0.122	−0.069	−0.117	−0.074	−0.069	−0.117
Ile	0.199	−0.006	−0.080	0.189	−0.017	0.052	−0.043	−0.069	−0.090
Leu	0.089	−0.074	−0.069	0.004	−0.027	−0.064	−0.006	0.073	−0.080
Lys	−0.111	0.004	−0.106	−0.122	0.052	−0.122	−0.090	0.010	−0.117
Met	−0.106	−0.101	−0.101	−0.101	−0.090	−0.111	−0.117	−0.111	−0.096
Phe	0.099	−0.074	−0.048	−0.069	−0.074	−0.122	−0.111	−0.074	−0.111
Pro	0.099	−0.117	−0.038	−0.122	−0.032	0.115	0.020	0.162	−0.122
Ser	−0.048	0.015	0.226	0.057	0.062	0.089	−0.006	0.004	−0.011
Thr	−0.074	0.068	0.147	0.010	0.052	0.078	−0.053	−0.059	−0.080
Trp	−0.038	−0.106	−0.111	−0.101	−0.106	−0.122	−0.106	−0.117	−0.106
Tyr	0.089	−0.011	−0.074	−0.122	−0.085	−0.111	−0.064	−0.069	−0.101
Val	0.210	0.078	0.004	0.352	0.178	0.462	0.004	0.010	−0.059

**Table 6:** Quantitative matrix (QM) for non-binding nonamers to HLA-DQ8.1.

AA	p1	p2	p3	p4	p5	p6	p7	p8	p9
Ala	−0.140	−0.140	−0.140	−0.140	−0.140	−0.140	−0.140	−0.140	−0.140
Arg	0.108	−0.140	0.217	0.108	−0.140	0.102	0.229	−0.140	0.108
Asn	0.181	−0.140	0.235	0.102	0.672	0.132	−0.140	0.793	0.138
Asp	−0.140	−0.140	−0.140	−0.140	−0.140	−0.140	−0.140	−0.140	−0.140
Cys	0.090	0.490	0.211	−0.140	0.860	0.187	0.260	0.751	0.126
Gln	0.138	−0.140	0.211	0.151	−0.140	0.145	−0.140	−0.140	−0.140
Glu	−0.140	−0.140	−0.140	0.145	−0.140	−0.140	−0.140	−0.140	−0.140
Gly	0.169	−0.140	−0.140	0.084	−0.140	0.169	0.302	−0.140	−0.140
His	0.126	−0.140	0.272	0.096	−0.140	0.193	−0.140	−0.140	−0.140
Ile	−0.140	−0.140	0.157	−0.140	−0.140	−0.140	−0.140	−0.140	0.066
Leu	0.102	0.611	0.199	0.108	0.854	0.132	0.211	0.842	0.084
Lys	0.175	−0.140	0.181	0.145	−0.140	0.126	0.235	−0.140	0.078
Met	−0.140	−0.140	−0.140	−0.140	−0.140	−0.140	0.302	−0.140	−0.140
Phe	−0.140	0.557	−0.140	0.060	−0.140	0.084	−0.140	−0.140	0.072
Pro	−0.140	0.587	−0.140	0.120	−0.140	−0.140	−0.140	−0.140	0.145
Ser	0.175	−0.140	−0.140	−0.140	−0.140	−0.140	−0.140	−0.140	0.138
Thr	0.138	−0.140	−0.140	−0.140	−0.140	−0.140	0.284	−0.140	0.138
Trp	−0.140	−0.140	−0.140	−0.140	−0.140	−0.140	−0.140	−0.140	−0.140
Tyr	−0.140	−0.140	−0.140	0.145	−0.140	0.132	−0.140	−0.140	−0.140
Val	−0.140	−0.140	−0.140	−0.140	−0.140	−0.140	−0.140	−0.140	0.169

## Data Availability

The data presented in this study are available in article.
